# Raloxifene and n-Acetylcysteine Ameliorate TGF-Signalling in Fibroblasts from Patients with Recessive Dominant Epidermolysis Bullosa

**DOI:** 10.3390/cells9092108

**Published:** 2020-09-16

**Authors:** Tania Aguado, Marta García, Adela García, Gemma Ferrer-Mayorga, Lucía Martínez-Santamaría, Marcela del Río, Luisa-María Botella, José-María Sánchez-Puelles

**Affiliations:** 1 Department of Molecular Biomedicine, Centro de Investigaciones Biológicas, Consejo Superior de Investigaciones Científicas, U-707 CIBERER, 28040 Madrid, Spain; tania.aguado@gmail.com; 2 Departament of Biomedical Engineering, Universidad Carlos III, 28911 Madrid, Spain; mgdiez@ing.uc3m.es (M.G.); adelagmartin@gmail.com (A.G.); lmsantam@ing.uc3m.es (L.M.-S.); mrnechae@ing.uc3m.es (M.d.R.); 3 Spanish Network of Research Groups on Rare Diseases (CIBERER) U714, 28911 Madrid, Spain; 4 Foundation of the Institute for Health Research, Jiménez Díaz Foundation, 28040 Madrid, Spain; 5 Department of Cancer Biology, Instituto de Investigaciones Biomédicas “Alberto Sols”, Consejo Superior de Investigaciones Científicas, Universidad Autónoma de Madrid, 28029 Madrid, Spain; gferrer@iib.uam.es

**Keywords:** epidermolysis bullosa, TGF-fibrosis, raloxifene, N-acetylcysteine, endoglin, smad, ALK1/5, drug repurposing

## Abstract

Recessive dystrophic epidermolysis bullosa (RDEB) is a severe skin disease caused by mutation of the *COL7A1* gene. RDEB is associated with high levels of TGF-β1, which is likely to be involved in the fibrosis that develops in this disease. Endoglin (CD105) is a type III coreceptor for TGF-β1 and its overexpression in fibroblasts deregulates physiological Smad/Alk1/Alk5 signalling, repressing the synthesis of TGF-β1 and extracellular matrix (ECM) proteins. Raloxifene is a specific estrogen receptor modulator designated as an orphan drug for hereditary hemorrhagic telangiectasia, a rare vascular disease. Raloxifene stimulates endoglin synthesis, which could attenuate fibrosis. By contrast, the antioxidant N-acetylcysteine may have therapeutic value to rectify inflammation, fibrosis and endothelial dysfunction. Thus, we present here a repurposing strategy based on the molecular and functional screening of fibroblasts from RDEB patients with these drugs, leading us to propose the repositioning of these two well-known drugs currently in clinical use, raloxifene and N-acetylcysteine, to counteract fibrosis and inflammation in RDEB. Both compounds modulate the profibrotic events that may ultimately be responsible for the clinical manifestations in RDEB, suggesting that these findings may also be relevant for other diseases in which fibrosis is an important pathophysiological event.

## 1. Introduction

Recessive dystrophic epidermolysis bullosa (RDEB) is a severe skin disease caused by loss-of-function mutations in the COL7A1 gene that encodes type VII collagen (C7). In keratinocytes and fibroblasts, C7 constitutes the main anchor that fibrils need to establish adhesion between the dermis and the epidermal basement membrane zone (BMZ). Hence, a deficiency in C7 provokes severe and recurrent blistering of the skin and other stratified epithelia, as well as scarring, and it seriously increases the risk of developing metastatic squamous cell carcinoma (SSC) [1]. Ex vivo gene therapy strategies for epidermolysis bullosa (as well as for junctional epidermolysis bullosa and RDEB) have been designed and they are already in clinical stages of testing. These approaches are based on the transplantation of retroviral-modified keratinocyte sheets, and they are producing promising results [2,3,4,5]. Indeed, recent grafting experiments provided evidence of the clinical potential of clonal gene therapy based on the excision of exons containing pathogenic mutations. Moreover, improved gene editing tools may make combined genetic and cellular approaches more feasible in the near future [6]. However, given the complexity and expense of these therapies, it is worth exploring complementary pharmacological strategies, such as the repositioning of approved drugs for new therapeutic indications, a strategy that streamlines the path towards clinical implementation. Repurposing capitalizes on prior investment (both in terms of time and money), while essentially annulling the risk of clinical activities to address new niches of urgent clinical need [7,8].

A few years ago, pioneering work revealed that the angiotensin II type 1 receptor antagonist, losartan, inhibited fibrosis in RDEB by effectively reducing TGF-β1 in cells in vitro, as well as TGF-β1 in circulation and in the skin of a RDEB mouse model [9]. The TGF signaling pathway influences diverse cellular processes including proliferation, cell fate, migration, apoptosis and extracellular matrix (ECM) remodeling, as has been extensively studied and reviewed elsewhere [10,11,12]. The membrane glycoprotein endoglin (CD105) acts as a coreceptor for the TGF-β1 and BMP families, and modulates the intracellular signaling activated by these factors, inhibiting TGF-β1 signaling, and influencing carcinogenesis and hematopoiesis [13,14]. Endoglin is found in the basal layer of the epidermis and hair follicles, and is expressed by the two main skin cells, keratinocytes and fibroblasts. Indeed, its overexpression in murine fibroblasts deregulates SMAD physiological/ALK signaling, repressing the synthesis of TGF-β1 and ECM proteins [15,16]. Raloxifene, and SERMs (specific estrogen receptor modulators) in general, are drugs that stimulate endoglin synthesis [17,18], and their effects in attenuating fibrosis suggest an involvement of endoglin and TGF-β1 in such events [19]. Alternatively, the antioxidant N-acetylcysteine (NAC) has recognized therapeutic value in inflammation, fibrosis and endothelial dysfunction, and promotes the synthesis of a wide number of genes by modulating signal-regulated kinase pathways, a concept that may be useful to treat certain degenerative diseases [20].

Recent transcriptomic analysis (RNAseq) of RDEB fibroblasts identified higher levels of basal reactive oxygen species (ROS), abnormal ECM deposition and a repression of antioxidant enzymes. These data suggest a redox imbalance may modulate disease severity, triggering fibroblast activation and ECM accumulation [21]. The involvement of the TGF-β pathway in modulating disease variability was elegantly demonstrated in RDEB fibroblasts from monozygotic twins, with canonical (Smads) and non canonical (MAPKs) TGF-pathways more active in the fibroblasts of the more affected twin. Considering the fibrotic hallmarks of RDEB identified in the literature, we designed an initial repositioning strategy based on the phenotypic screening of COL7A1-deficient fibroblasts from RDEB patients and focused on these potential biomarkers. Through this strategy, we propose the repositioning of two well-known drugs that have been in clinical use for decades: a SERM ligand that enhances serum endoglin, raloxifene; and the antioxidant NAC commonly used as anti-inflammatory antioxidant agent, and that is involved in the negative regulation of NF-kB signaling. Both compounds modulate complex profibrotic events that may ultimately be responsible for multiple clinical manifestations in RDEB.

## 2. Results

### 2.1. Weaker TGF-β1 Expression in Human RDEB Fibroblasts Following the Pharmacological Stimulation of Endoglin Expression

TGF-β1 is closely associated with fibrosis in several organs [9,11,12] and its induction is an important facet of RDEB fibrosis. To examine this relationship, RDEB fibroblasts from three different patients were cultured in the presence of raloxifene, NAC or losartan, and the expression of endoglin and TGF-β1 (total and active) was assessed by ELISA. The doses used for each of these compounds were based on those used previously in the literature [9,17,22]. As the TGF/Alk/Smads signaling pathway is the same in normal and RDEB fibroblasts, we assumed that the response of normal fibroblasts to these drugs would be the same, as shown elsewhere [9]. However, this response would be expected to be stronger in RDEB fibroblasts given the higher levels of TGF.

**Figure 1:**
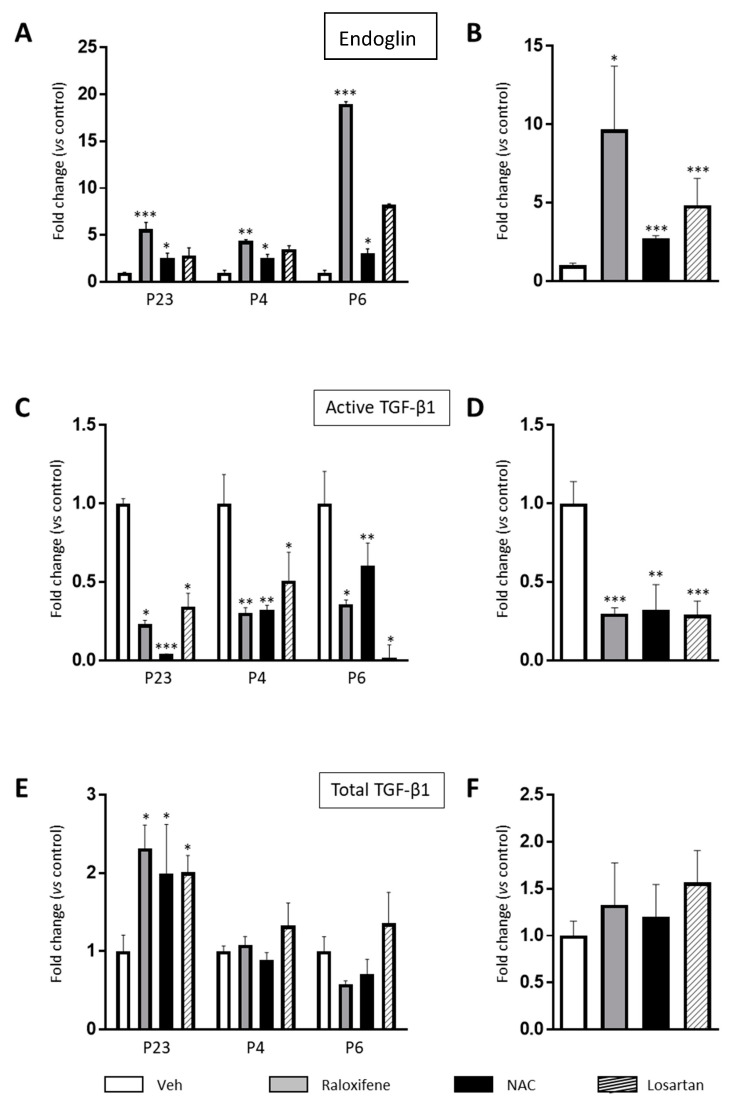
Raloxifene and N-acetylcysteine (NAC) modulate endoglin and TGF-β1 protein levels. The effect of raloxifene (0.2 nM), NAC (100 μM) and losartan (10 µM) on endoglin and on the active and total TGF-β1 was assessed by ELISA at 48 h in serum starved fibroblast from recessive dystrophic epidermolysis bullosa (RDEB) patients. (**A**,**C**,**E**) represents the change in endoglin and TGF-β1 in fibroblasts from the three patients (P23, P4 and P6) The mean change relative to the controls is shown in three independent experiments for each patient. (**B**,**D**,**F**) indicate the mean change in the three patients. The differences were statistically significant according to the Student’s *t*-test: * *p* < 0.05; ** *p* < 0.01; *** *p* < 0.001.

Raloxifene, NAC and losartan each increased the amount of soluble endoglin detected in the fibroblasts from the three RDEB patients by three to 10-fold, a direct reflection of the increase in membrane endoglin (Figure 1A,B). This increase in endoglin levels was coincident with a significant decrease in the active TGF-β1 fraction (Figure 1C,D). However, neither raloxifene nor NAC appeared to affect the total TGF-β1 (Figure 1E,F), although there was significantly more total TGF-β1 in fibroblasts from one patient, P23, than in those from the other two patients after exposure to each of the three drugs. Therefore, exposing RDEB fibroblasts from three patients to the endoglin-stimulating drug raloxifene or the antioxidant NAC provoked an elevation in endoglin protein (Figure 1B), while diminishing the active TGF-β1 fraction (Figure 1D).

### 2.2. Raloxifene and n-Acetylcysteine Regulate Fibrosis Associated Biomarkers

**Figure 2:**
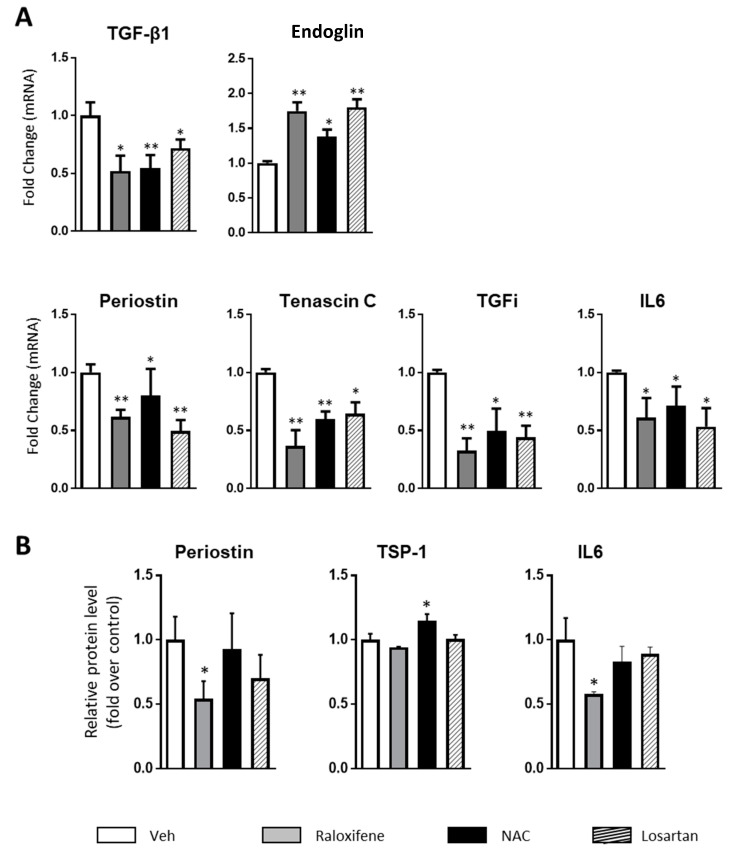
Raloxifene and NAC regulate fibrosis-associated biomarkers. RDEB fibroblasts from the three patients were cultured for 48 h in the presence of raloxifene (0.2 nM), NAC (100 μM), losartan (10 µM) or the vehicle alone (Veh). (**A**) RT-qPCR (Reverse Transcription followed by quantitative PCR) analysis of TGF-β1 and endoglin RNA expression, and of the transcripts encoding fibrosis and inflammation associated genes. The graphs show the mean change relative to fibroblasts exposed to the vehicle alone. (**B**) Secreted periostin, TSP-1 and IL-6 protein in medium conditioned by RDEB fibroblasts treated with raloxifene, NAC or vehicle for 48 h as measured by ELISA. The graphs show the mean of change relative to fibroblasts exposed to the vehicle alone. Differences were statistically significant according to the Student’s *t*-test: * *p* < 0.05; ** *p* < 0.01.

The target genes activated by TGF-signaling are responsible for a wide range of cellular processes, including proliferation, differentiation, migration and apoptosis. In relation to RDEB, this signaling influences ECM remodeling and inflammation, leading to fibrosis and chronic inflammation [9,10,12]. Since raloxifene, NAC and losartan upregulate endoglin in conjunction with a decrease in the active TGF-β1 protein, we explored endoglin and TGF-β1 mRNA expression in fibroblasts from the same three patients (Figure 2A), as well as that of some representative RDEB biomarkers like periostin, thrombospondin (*TSP*-1), Transforming Growth Factor Inhibitor (TGFI), Tenascin C and IL-6, the latter the main inflammatory biomarker altered in different models of RDEB [9,21] (Figure 2A). In line with the results obtained at the protein level, treatment with the three drugs diminished TGF-β1 mRNA while augmenting that encoding endoglin (see Figure 2A for the average of the three patients). Interestingly, exposing the RDEB fibroblasts to raloxifene, NAC or losartan significantly dampened the expression of the mRNA transcripts encoding the selected TGF-β1 targets associated with fibrosis and inflammation in RDEB. In addition, the high levels of IL-6 protein in the RDEB fibroblasts were also significantly diminished by raloxifene treatment, and there was a tendency towards a reduction following exposure to NAC and losartan. The TGF-β1 target proteins like TSP-1 and periostin are considered a hallmark of RDEB and thus, they were also assessed (Figure 2B). Raloxifene significantly diminished the expression of these relevant RDEB biomarkers in all three of the patients tested, whereas NAC and losartan produced distinct responses of the ECM proteins, while inducing a relevant inhibition of TGF-1 expression. It was particularly notable that NAC treatment provoked a significant increase in TSP-1 protein levels. It is known that oxidative stress-induced attenuation of thrombospondin-1 expression occurs in primary rat astrocytes. This inhibitory effect is blocked by NAC, in good agreement with our data [23]. Thus, raloxifene and NAC appeared to produce potentially antifibrotic effects in RDEB human fibroblasts, dampening the excess TGF-1 expression and that of its downstream biomarkers.

### 2.3. Raloxifene and NAC Modulate the Canonical TGF-β1/Smad Signaling Pathway

**Figure 3:**
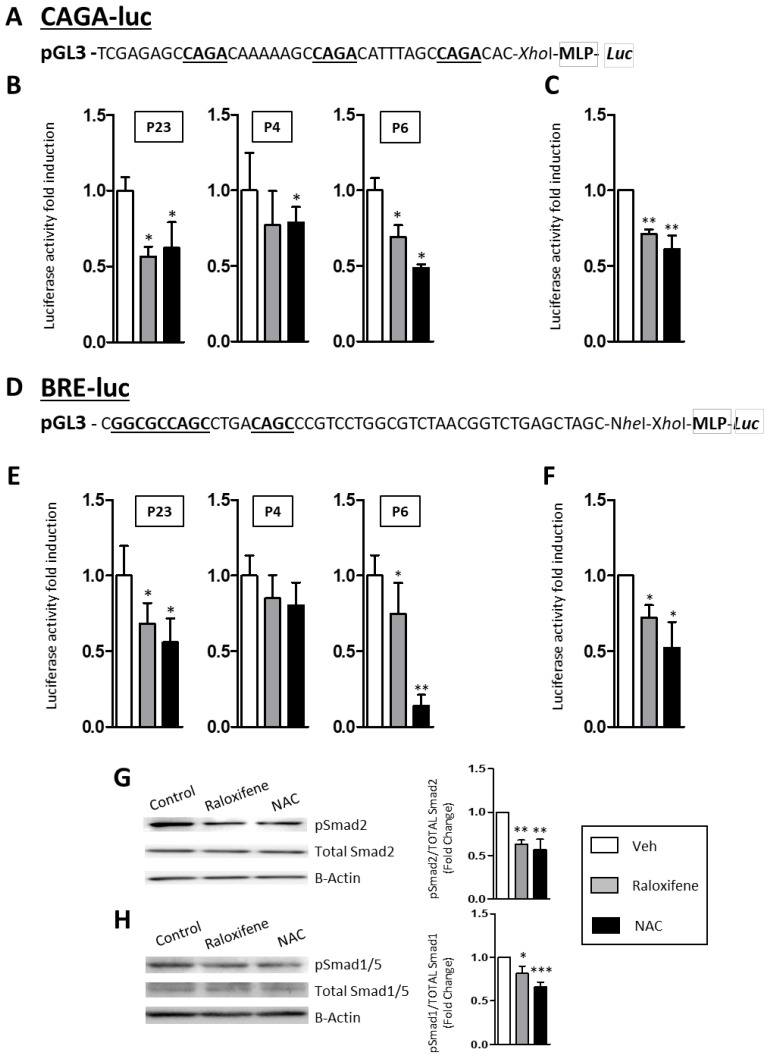
Raloxifene modulates TGF-β1 signaling. Human fibroblasts from RDEB patients were transiently transfected with the CAGA-luc (**A**,**B**) and BRE-luc (**D**,**E**) reporter vectors in which the SBE (Smad Binding Elements) sequences in the promoter of the reporters is underlined. Luciferase assays were performed on the fibroblasts from the three patients. Panels (**B**,**E**) show the mean induction in the three patients individually, while (**C**,**F**) is the average of the three patients altogether. Luciferase activity was measured in untreated cells or cells exposed to raloxifene or NAC for 48 h, in the presence of TGF-β1 during (**B**) the last 24 h, or (**E**) 3 h. (**G**,**H**), Western blot analysis of p-Smad2 (**G**) and p-Smad1/5 (**H**) protein in cells treated with raloxifene, NAC or the vehicle alone during 48 h in the presence of TGF-β1 during (**G**) the last 24 h or (**H**) 3 h. Total Smad2 or Smad1/5 were used as controls and β-Actin was used to confirm similar loading between the samples. Representative Western blots are shown and the quantification (mean ± SEM) of the three patients are shown. Differences were statistically significant according to the Student’s *t*-test: * *p* < 0.05; ** *p* < 0.01; *** *p* < 0.005.

The mechanism by which Raloxifene and NAC dampened the expression of TGF-β1 in RDEB fibroblasts was investigated using different transfection vectors associated with distinct elements of the canonical TGF-β1/Smad signaling pathway. Thus, a Smad2/3 luciferase construct (pAd.CAGA(12)-luc) [24] was used as a TGF-β1-Alk5/Smad2/3 reporter, as was a BMP (Bone Morphogenetic Protein)/luciferase reporter (Id1-BRE-*Luc*) that contained the *Bmp2* response element (12x*GCGC*) [25] and served as a TGF-β1-Alk1/Smad1/5 reporter (see Figure 3 for a scheme of the reporters), both these constructs (BRE-LUC and CAGA-Luc) containing SBE (Smad Binding Elements) sequences (underlined in Figure 3). Fibroblasts from the three patients (P23, P4 and P6) were transfected with these TGF-β1/Smad reporters and then exposed to raloxifene, NAC and losartan. When the treatments were normalized to the cells exposed to the vehicle alone, and to those treated with TGF-β1 as a stimulated control, there was a significant decrease in the mean luciferase signal obtained from the TGF-β1-Alk5/Smad2/3 construct in the fibroblasts from the three RDEB patients (Figure 3A,B and Figure S1 for losartan). Likewise, a decrease in luciferase activity was also observed when RDEB fibroblasts harboring TGF-β1-Alk1/Smad1/5 were examined (Figure 3C,D and Figure S1). Therefore, both the Smad2/3 and Smad1/5 pathways appeared to be significantly downregulated when RDEB fibroblasts were exposed to raloxifene, NAC and losartan. Moreover, the activity of these TGF-β1 pathways was also analyzed in Western blots (Figure 3G,H), evaluating the amount of pSmad2 relative to the total Smads to monitor the TGF-β1-Alk5/Smad2/3 pathway, while the amount of pSmad1/5 relative to actin was assessed as a measure of the TGF-β1-Alk1/Smad1/5 activity. Activation of both TGF-β pathways was downregulated by treatment with raloxifene and NAC, with less pSmad2 in Western blots reflecting a downregulation of the TGF-β1-Alk5/Smad2/3, and less pSmad1/5 reflecting that of TGF-β1-Alk1/Smad1/5 (Figure 3G,H). Interestingly, there was stronger basal pSmad1/5 activity in RDEB fibroblasts in the absence of exogenous TGF–β stimulation (Figure 3G,H).

### 2.4. Raloxifene and NAC Limit the Contractility of Fibroblasts from RDEB Patients in the Collagen Gel Contraction Assay

**Figure 4:**
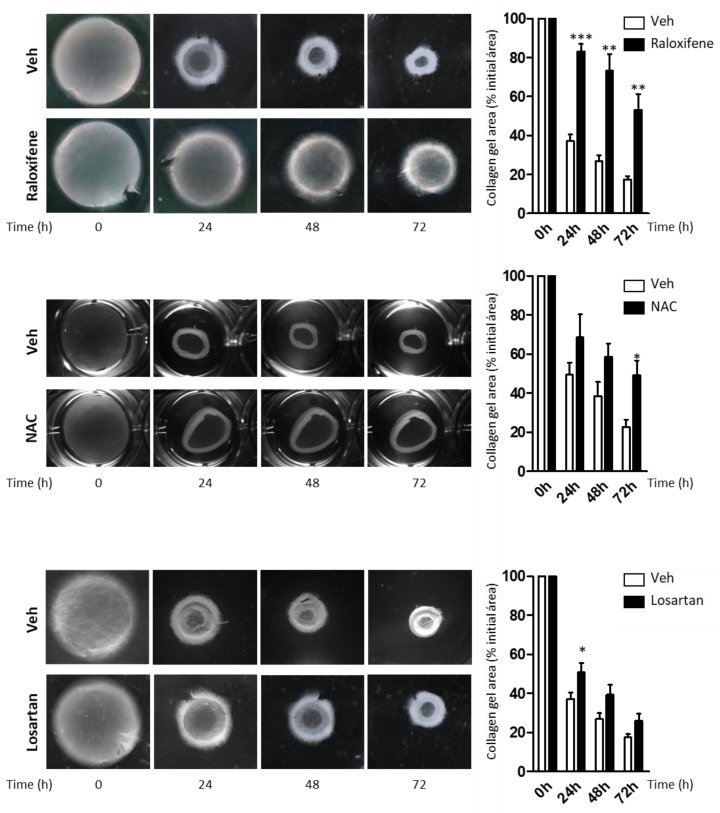
Effect of raloxifene an NAC on contraction in a collagen gel assay. Fibroblasts from RDEB patients were embedded in collagen gels in the presence of raloxifene (5 μM), NAC (1 mM), losartan (10 µM) or the vehicle alone (Veh), and the gel area was measured at the times indicated. Left, representative stereomicroscope images of the collagen gels. Right graphs show the contraction of collagen gels at the times indicated in reference to the surface area at time 0, represented as the mean contraction relative to Veh of three independent experiments (mean ± SEM): * *p* < 0.05; ** *p* < 0.01; *** *p* < 0.005.

The ECM remodeling that occurs in patients with RDEB as fibrosis progresses stiffens the tissues. To assess how raloxifene and NAC affected ECM remodeling and tissue biomechanics, limiting RDEB fibroblast contractility, functional tests of collagen gel contraction lattices were performed [26], employing losartan as a control for fibroblast contractility [9]. In line with previous data [27], no differences in the morphology of the fibroblasts isolated from control donors and RDEB patients were observed in 2D culture. However the expression of markers such as α-smooth muscle actin (α-SMA) and TGFBi did vary (Supplementary Figure S2). Accordingly, exposure of these cells to NAC, raloxifene or losartan effectively inhibited RDEB fibroblast contractility (Figure 4), which reflected a reduction in the proportion of activated fibroblasts.

Furthermore, to demonstrate ECM differences in RDEB vs. control fibroblasts, we performed immunofluorescence studies to assess the distribution of two markers in control donor and RDEB fibroblasts in 2D culture: α-smooth muscle cell actin (α-Sma) and TGFBi (Supplementary Figure S2). The proportion of cells that expressed α-SMA and TGFBi in the two different control samples and two RDEB patients were assessed in 10 different fields, and a significant increase in α-SMA and TGFBi was evident in the fibroblasts from RDEB patients, reflecting the distinct ECM response of these cells.

### 2.5. The Presence of Active TGF-β Peptide in the Serum of 22 RDEB Patients Indicates It May Represent a Disease Biomarker

**Figure 5:**
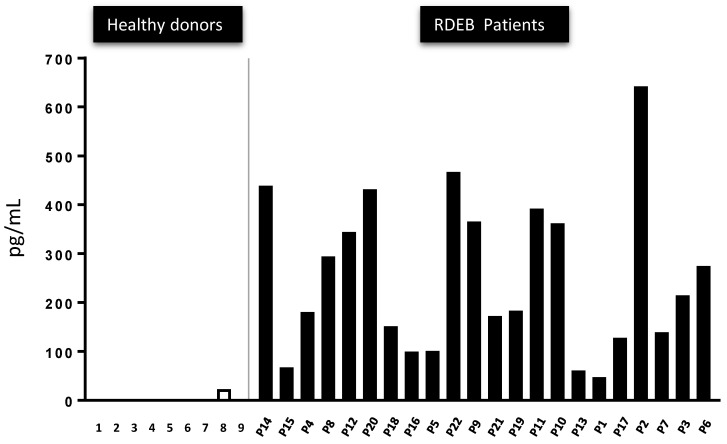
The active TGF-β1 peptide in the serum of 22 RDEB patients relative to the healthy control individuals. ELISA measurement of the active TGF-β1 peptide showing aberrant high levels of this peptide and its absence (detectable) in healthy individuals.

Human RDEB is associated with enhanced TGF-β1 expression and activity, as also evident in mouse models replicating this disorder [9,11,12]. The amount of total TGF-β1 and the active TGF-β1 fraction was evaluated in serum from 23 RDEB patients and compared with that in nine healthy controls. On average, and as expected from the literature, there appeared to be more total TGF-β1 in RDEB patients than in the controls, although the differences detected were not necessarily statistically significant (Supplementary Figure S3). However, very different values were obtained when the active TGF-β1 fraction was measured (Figure 5). While virtually no active TGF-β1 was detected in the controls, significant amounts of active TGF-β1 were detected in all RDEB patients. The profile of the 23 patients in our cohort is summarized in Supplementary Table S1.

## 3. Discussion

RDEB is both a skin disease and a fibrotic disorder in which changes in TGF-β1 activity contribute significantly to its progression [1]. In other genetic disorders with increased ECM deposition, preclinical studies and phase I–III trials have aimed to downregulate TGF-β1 using antibodies or soluble TGF-β1 receptors as scavengers, or by blocking the activation of latent TGF-β1. However, these efforts have had limited success [28,29,30]. Moreover, most of the agents used in these approaches are not currently employed in clinical practice due to their high cost, limited efficacy or problems of safety provoked by multiple associated side-effects, a phenomenon that should be of no surprise given the many and varied roles of TGF-β1 signaling [30]. By contrast, the first efforts to develop a specific therapy for RDEB were based on gene therapy, reintroducing the deficient C7 gene into the skin through the use of an attenuated virus, cell approaches or even using recombinant protein [6]. Nevertheless, these attractive strategies still require further clinical development and, as such, they can only be considered as medium or long-term applications in humans. Moreover, the high cost of such therapies per patient is another barrier to their uptake by healthcare systems [29].

An alternative strategy that will more rapidly reach clinical phases is the repurposing of known drugs initially used for other clinical purposes; drugs whose safety profile, side-effects and pharmacodynamics are already well-established in clinical settings. Repurposing is a strategy applied previously in RDEB fibroblast cultures and in an animal model of this disease, showing that losartan effectively reduces the TGF-β1 and IL-6 protein in RDEB cells in vitro, and in the skin and circulatory system of the RDEB mouse model [9]. However, losartan can provoke hypotension, which limits its use in RDEB patients as many of them suffer from anemia and hypotension. A strong chain of translatability has been suggested for drugs that ameliorate molecular hallmarks of disease in phenotypic screens, such as those using human COL7A1-deficient RDEB fibroblasts [31]. In fact, it was predicted that human RDEB fibroblasts could help to identify compounds that modulate or prevent disease progression in RDEB patients to a greater extent than losartan in mice. As such, here we performed a phenotypic screening of human COL7A1-deficient fibroblasts with the endpoint of lowering TGF-β1 protein, and with the aim of limiting fibrosis and inflammation.

In the first place, we studied raloxifene as a candidate drug to alleviate RDEB on the basis of data linking the stimulation of endoglin to the negative modulation of Alk/Smad pathways and a decrease in TGF-β1 [14,18,30], a master regulator of fibrosis. Raloxifene was designated as an orphan drug by the EMA and FDA in 2010, and it is now being used to treat hereditary hemorrhagic telangiectasia (HHT) [18]. This use of raloxifene to combat bleeding is based on its capacity to upregulate endoglin synthesis and to modulate Alk1/Smad signaling in endothelial cells [30], with both endoglin and ALK1 known to be haploinsufficient in HHT. Further studies into the negative modulation of the Alk5/Smad pathway by endoglin indicated it was associated with the downstream inhibition of TGF-β1 synthesis and of its targeted ECM proteins, endowing endoglin the attractive potential to attenuate fibrosis [32,33]. Remarkably, raloxifene has also been repositioned as an anticancer drug in breast cancer [34,35,36], hepatoma [36,37] and prostate cancers [38,39], and this antineoplastic profile of raloxifene may represent a relevant added value for RDEB, since patients suffer a very aggressive form of skin cancer at advanced stages of the disease.

As signaling effectors, growth factors and proinflammatory events elevate ROS levels, the widely used antioxidant NAC has pharmacological properties that reduce ROS in pathological processes related to endothelial dysfunction, inflammation, fibrosis, invasion, cartilage erosion, etc. Accordingly, NAC promotes cell survival by activating the extracellular signal-regulated kinase pathway, a concept useful for treating certain degenerative diseases [21,40,41]. Considering these phenomena, along with its safe clinical use over decades, NAC was also investigated for its capacity to attenuate the RDEB phenotype. We found that the high levels of active TGF-β in RDEB fibroblasts from three different patients, a hallmark of the disease, were diminished following exposure to either raloxifene or NAC, in direct contrast to the increase in endoglin expression. This accumulation of active TGF-β1 is likely to be the result of altered dermal tissue architecture that releases the matrix-bound protein, and of inflammation following tissue damage. We did consider the use of these two drugs in combination, yet these experiments did not produce conclusive data, probably due to the limited number of patients studied. Moreover, while both these drugs have been in clinical use for decades, their safety profiles when used in combination would have to be reassessed in well-designed clinical trials, partially reducing the attractiveness of the approach used here.

Based on these data, we collected serum from 22 RDEB patients and found a relative increase in the total circulating TGF-β to aberrantly high levels in all the samples relative to healthy controls in which active TGF-β1 peptide was practically undetectable. Although levels of active TGF-β1 can be seen in different diseases, and may reflect processes such as wound-healing, active inflammation and autoimmune events, we propose here that active TGF-β1 might serve as a biomarker in the follow up of patients with RDEB; a possibility that would have to be evaluated in more detailed clinical trials under strict regulatory rules. Disease progression to carcinogenesis has been reviewed previously [42] and, in the case of RDEB, several studies have related age with the increase of SCC in patients with RDEB; this type of cancer representing the most serious complication of EB in adults, especially those with severe generalized RDEB in whom the median age of SCC onset is 30 years [43,44]. However, due to the heterogeneity in the ages of our patients, we cannot draw any correlation between TGF1 levels and the appearance of SCCs and more focused studies must be designed to study this in more detail.

Abnormally high levels of IL-6 are consistently reported in cell models and patients with RDEB. Raloxifene and NAC markedly reduced IL-6 mRNA and protein expression in the fibroblasts from the three RDEB patients tested. In addition, IL-6 is induced by TGF-β1 stimulation in many cell types, including fibroblasts, and it contributes to monocyte recruitment and inflammation in the stroma [45,46,47]. Increased IL-6 expression mediated by TGF-β1 contributes to the differentiation of fibroblasts into myofibroblasts, which in turn amplifies the proinflammatory and profibrotic effects of TGF-β1 [46]. Moreover, our results are consistent with data from monozygotic twins with distinct disease phenotypes. As such, TGF-β1 protein release was enhanced around two-fold and higher levels of IL-6 were evident in the fibroblasts of the twin with the more severe clinical manifestations [48]. The most prominent response of dermal fibroblasts to TGF-β1 activity is increased deposition of ECM components [49]. Both, raloxifene and NAC modulate TGF-β1 driven matrix remodeling by dampening the transcription of tenascin C (TNC), periostin and TGFI, all of which influence ECM integrity. TNC is a relevant marker of fibrosis, an ECM protein involved in several physiological and pathological conditions, and it is induced by the TGF-β1/Alk5/Smad2/3 pathway [50,51]. Although losartan, raloxifene and NAC significantly diminish the active TGF-β1 available, only raloxifene also lowers periostin, Tsp1 and IL-6. Periostin is upregulated by TGF-β1 in several fibrotic diseases [52,53], and only recently has an RNA-Seq strategy revealed it to be a putative systemic biomarker in RDEB [21]. Elevated serum periostin is associated with disease progression and the severity of pulmonary fibrosis [54], colorectal cancer [55,56] and systemic sclerosis [57]. The overexpression of periostin and TNC is induced by skin damage, promoting the activation of fibroblasts to repair the wound, and their pathogenicity has been associated with chronic inflammation, fibrosis and cancer [58,59,60].

Although TGF-β1 plays a dominant role in the initiation and progression of renal fibrosis, its effector Smad proteins can have distinct and even opposing regulatory effects in fibrosis [12]. We also show here that drugs that augment endoglin negatively modulate the canonical TGF-β1 pathway (TGF/Alk/Smad). Therefore, endoglin can fulfil a dual role in canonical Smad signalling in endothelial cells due to the specific expression of ALK1 [33,61]. In the case of the Alk5/Smad2/3 pathway, ubiquitous in all cells, TGF-β1 binds to the receptor complex and through phosphorylation of the type I receptor in fibroblasts, to ALK5. In turn, Smad2/3 phosphorylation provokes the upregulation of genes involved in ECM synthesis, inflammation and even in the upregulation of TGF-β1 in an autocrine feedback loop [61]. This phosphorylation of Smad2/3 would evoke a vicious cycle of ECM overproduction, more TGF-β1, inflammation, and rigidity of fibroblasts and the dermis. However, this pathway would be negatively modulated in the presence of endoglin, which acts as a dose-dependent modulator. By upregulating this natural negative modulator, endoglin would appear to provide a sensitive and effective mechanism to bypass the undesired pathophysiological effects of TGF-β1 pathway.

**Figure 6:**
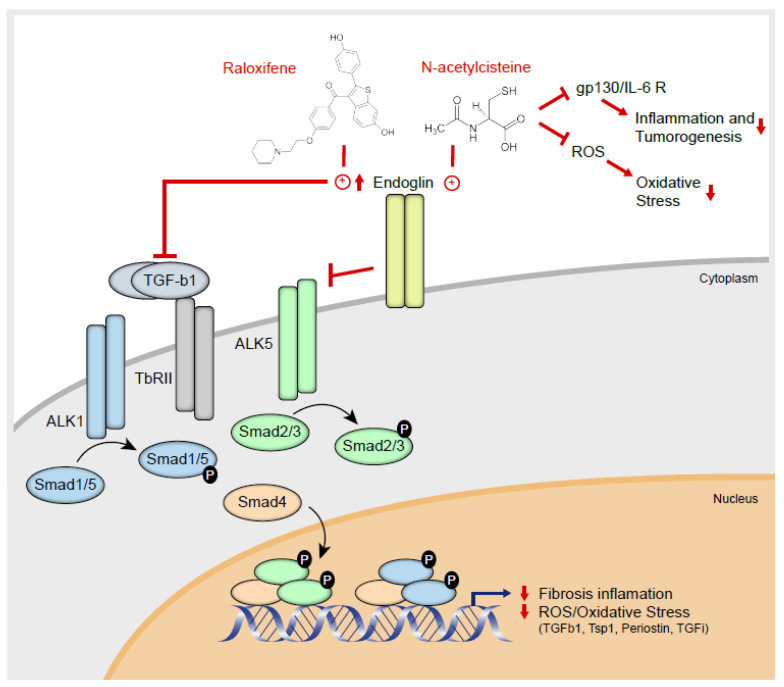
Novel therapies for RDEB based on the dysregulation of upstream extracellular matrix (ECM) mechanisms. Mechanistic mode of action of raloxifene and NAC, activating endoglin and repressing TGF-β1 signaling through the ALK/Smad pathways, thereby dampening fibrosis, inflammation and the effects of reactive oxidative species (ROS). The physiological role of ROS/oxidative stress in RDEB has been defined previously [21].

Signals are transmitted in the canonical TGF-β1 pathway via Alk1/Smad1/5 phosphorylation and by activating the inhibitor of differentiation (Id) genes, provoking proliferation and de-differentiation. These events take place as part of the epithelial-mesenchymal transition (EMT), implicating this Smad cascade in tumorigenesis and developmental pathways. In fact, the wounds and scars in RDEB ultimately progress to SCC. Interestingly, constitutive phosphorylation of Smad 1/5 was detected in RDEB fibroblasts from the three patients tested here, in the absence of external TGF-β1 stimulation, indicating that there is constitutive activation of this pathway in this rare disease. Since excessive secretion of active TGF-β1 is a hallmark of RDEB, the endogenous levels of this factor may be responsible for the phosphorylation of Smad1/5, and for the stimulation of genes involved in cell proliferation and de-differentiation. Interestingly, stimulation of endoglin production by raloxifene, NAC and losartan also diminishes Smad1/5 phosphorylation. Thus, we propose a model (Figure 6) wherein the downregulation of TGF-β1/Smad signaling, in which endoglin overexpression may participate as a coreceptor, ameliorates disease symptoms through a direct interaction with SERM receptors. This mechanism opens up new potential scenarios for the pharmacological treatment of RDEB and it deserves further study, not only in this but in other malignancies involving fibrosis and inflammation.

The identification of new, hitherto unknown mechanisms of action for marketed medicines is the first step in assessing their potential reorientation for new therapeutic indications. By defining the pharmacological effects of raloxifene and NAC on the exaggerated TGF signaling mediated by SMAD proteins in primary cultures of human RDEB fibroblasts, this study further defines the molecular mechanisms that convert RDEB into a systemic inflammatory and fibrotic disease (Figure 6). HHT biology establishes a model by which SERMs lead to endoglin overexpression by endothelial cells. Therefore, these results constitute a rational basis to assess the potential role of SERM receptors in RDEB and also their potential clinical use to combat the exacerbated inflammation caused by the emergent viral infection consequence of the SARS-CoV-2 coronavirus [62].

In addition, the common genetic signature in fibroblasts of the three patients shares some similarities with fibrotic diseases, suggesting that new pharmacological approaches may offer relief from symptoms using the widely used antioxidant NAC, such as those involved in addressing oxidative status [20,27,63].

In summary, evidence is presented here that favors the use of raloxifene and NAC as repurposed drugs to attenuate RDEB symptoms, the most aggressive form of this rare DEB disease. Given their extended and safe clinical use in adults, raloxifene and NAC seem to be ideal repurposed drugs to be used as disease-modifying therapies for RDEB.

## 4. Methods

### 4.1. Human Subjects

Skin biopsies were obtained from patients after gaining approval from the ethics committee of the collaborating hospital and receiving the patients’ informed consent. All clinical procedures were approved by the ethics committees of La Paz University Hospital codes: HULP PI-1359 and HULP PI-1595 and conducted in accordance with the declaration of Helsinki. All samples were obtained after corresponding written informed consents. The fibroblasts and sera from RDEB patients were stored and registered in the National Registry of Biobanks (Reference: C. 0002937), and the cells were incorporated into a biobank located at the Community Centre for Blood and Tissues of the Principality of Asturias (National Register of Biobanks with Reference: C.0002961). Preclinical protocols were registered with the Ethics Committee of the Centre for Biological Research Margarita Salas and approved by the ethics subcommittee of the Consejo Superior de Investigaciones Científicas.

### 4.2. Culture of Primary RDEB Patient Fibroblasts

Human dermal fibroblasts were isolated from skin biopsies obtained from both healthy donors and RDEB (sev-gen RDEB) patients [64]. Primary human RDEB and healthy donor fibroblasts were cultured on plastic in Dulbecco’s modified Eagle’s medium (DMEM) containing 10% fetal calf serum. The cells were cultured at 37 °C in a humid atmosphere containing 5% CO_2_, and the medium was changed every two days [65]. All experiments in this study were done on fibroblast cultures from passage 5 to 8.

### 4.3. Primary RDEB Patient Fibroblasts Transfection and Reporter Assay

Transient transfections of RDEB patient fibroblasts were carried out in P-24 plates using 1 µg of DNA from the BRE-luc or CAGA-luc reporters [66,67] and a commercial transfection reagent (Superfect; QIAGEN). After transfection, the cells were incubated in the presence or absence of NAC or raloxifene for 24 h. The cell lysate was then assayed for luciferase expression using the Promega luciferase assay kit and measured in a TD20/20 luminometer (Promega, Madison, WI, USA). Relative luciferase activity was expressed in arbitrary units with respect to the total protein measured using a standard Bradford assay, according to the manufacturer’s instructions (Bio-Rad, Herts, UK). Transfections were performed in triplicate and repeated in at least in three independent experiments. Representative experiments are shown in the figures. For treatment with TGF-β1, it was added at 1 ng/mL for 3 h to stimulate the ALK1/Smad1/5 pathway or 10 ng/mL for 24 h to stimulate the ALK5/Smad2/3 pathway.

### 4.4. Collagen Gel Contraction Assay

Assays were performed as described previously [27], preparing the collagen gels by mixing fibroblasts with PureCol bovine type I collagen (Advanced Biomatrix, San Diego, CA, USA), 5 × DMEM, 0.1 M NaOH and distilled water (final concentrations: 1.7 mg/mL PureCol, 1 × DMEM and 3 mMNaOH). The mixture was seeded in 24-well cell culture plates and allowed to polymerize. After 24 h, and to initiate gel contraction (time 0), gels were released from the 24-well plates and transferred to 6-well plates containing culture medium with raloxifene (5 μM), NAC (1 mM), losartan (10 μM) or the vehicle alone. At the times indicated, images were taken with an E4500 digital camera (Nikon, Tokyo, Japan) mounted on a MZ6 modular stereomicroscope (Leica, Wetzlar, Germany), and the gel area was measured with ImageJ (National Institutes of Health, Bethesda, MD, USA). Images were processed using Adobe Photoshop CS6 (San Jose, CA, USA) and the experiments were performed in triplicate.

### 4.5. RNA Extraction and Relative Quantification of mRNA

RNA was isolated using the Direct-zol RNA MiniPrep Kit (ZymoResearch, Los Angeles, CA, USA) from RDEB fibroblasts washed with PBS, scraped off the plates and spun down. The pellet was treated with the Tri Reagent and homogenized for five min at room temperature before using the MiniPrep Kit as per instructions. The quality and concentration of the total RNA was evaluated by measuring the absorbance at 260, 230 and 280 nm on an ND-1000 spectrophotometer (NanoDrop Technologies, Wilmington, DE, USA). In all cases, the expected 260/280 (~2.0) and 260/230 (2.0–2.2) ratio values accepted for pure RNA were obtained.

The PCR conditions consisted of an initial activation at 50 °C for two min, followed by 40 cycles of 95 °C for 5 s, and 60 °C for 30 s in LightCycler 480 Real-Time PCR System (Roche, Pleasanton, CA, USA). The *C_t_* (threshold cycle) value for each primer was normalized to that of RNU6. To assess gene expression, cDNA was synthesized with the transcriptor first strand cDNA synthesis Kit (Roche) and the genes studied were amplified using the following PCR conditions: 40 amplification cycles of 95 °C for 10 s, 60 °C for 30 s and 72 °C for 30 s. ACTB was used as a reference gene for normalization and the real-time PCR reactions were performed in triplicate using the PerfeCTa SYBR Green SuperMix (Quanta BioSciences, Houston, TX, USA) and the FastStart Universal SYBR Green Master (Roche) for gene expression. The expression of each gene was determined by the relative standard curve method and the primer sequences for target genes were obtained from the Universal ProbeLibrary Assay Design Center (https://qpcr.probefinder.com/organism.jsp).

### 4.6. Western Blotting

Protein extracts were prepared with RIPA buffer, and equal amounts of protein were resolved by SDS-PAGE and then transferred to nitrocellulose membranes that were probed with antibodies against pSmad2 (1/200 dilution, Cell Signaling), total Smad (1/200 dilution, Cell Signaling), pSmad 1/5 (1/200 dilution, Cell Signaling), total Smad 1/5 (1/200 dilution, Cell Signaling) and a β-actin-HRP antibody (1:50,000 dilution; Sigma) used as a loading control. Densitometry was performed using ImageJ software and at least three independent experiments were performed for each condition.

### 4.7. Immunofluorescence Staining

For immunofluorescence detection of a-SMA and TGFBi in fibroblasts, cells grown on glass coverslips were fixed in methanol/acetone (1:1) for 10 min at −20 °C. After washing three times in PBS and once in PBS with 3% BSA (Bovine Serum Albumine) (Sigma-Aldrich, St. Louis, MO, USA) for 30 min, cells were incubated with 1A4 Monoclonal Anti-Actin, α-Smooth Muscle (Sigma-Aldrich, St. Louis, MO, USA), at 1:200 dilution and TGFBI/BIGH3 Polyclonal antibody (PROTEINTECH, Chicago, IL, USA) at 1:100 dilution. Secondary antibody (AlexaFluor488, Invitrogen, Carlsbad, CA, USA) was used at 1:1000 dilution. After the final washing step in PBS, preparations were mounted using Mowiol (Hoechst, Somerville, NJ, USA) mounting medium and DAPI 20 mg/mL (Sigma-Aldrich, St Louis, MO, USA) for nuclei visualization.

### 4.8. Statistical Analysis

The results are expressed as the mean ± SD and the statistical significance was assessed using a two-tailed unpaired Student’s *t* test with GraphPad Instat3 (La Jolla, CA, USA), unless otherwise specified. Differences were considered significant when *p* < 0.05: * *p* < 0.05, ** *p* < 0.01 and *** *p* < 0.001.

## Supplementary Materials

The following are available online at https://www.mdpi.com/2073-4409/9/9/2108/s1, Figure S1: Losartan modulates TGF-β1 signalling, Figure S2: Characterization of control donor and RDEB fibroblasts in 2D culture, Figure S3: The total TGF-β1 peptide in the serum of 22 RDEB patients relative to the healthy control individuals. Table S1: Sex, gene/protein mutation, clinical phenotype, severity and SCCs and age (biopsy taken) of the 22 RDEB patients used in the analysis of the Protein levels of active TGF-β1 peptide in their sera.
