# Supplementary material for: Raloxifene and n-Acetylcysteine Ameliorate TGF-Signalling in Fibroblasts from Patients with Recessive Dominant Epidermolysis Bullosa
Source: Cells. 2020 Sep 16;9(9):2108. doi: 10.3390/cells9092108 (PMC7565802; doi:10.3390/cells9092108)
Supplement: Supplementary file 1 [file cells-09-02108-s001.zip › cells-911863 supplementary/cells-911863 supplementary proofreading.docx]

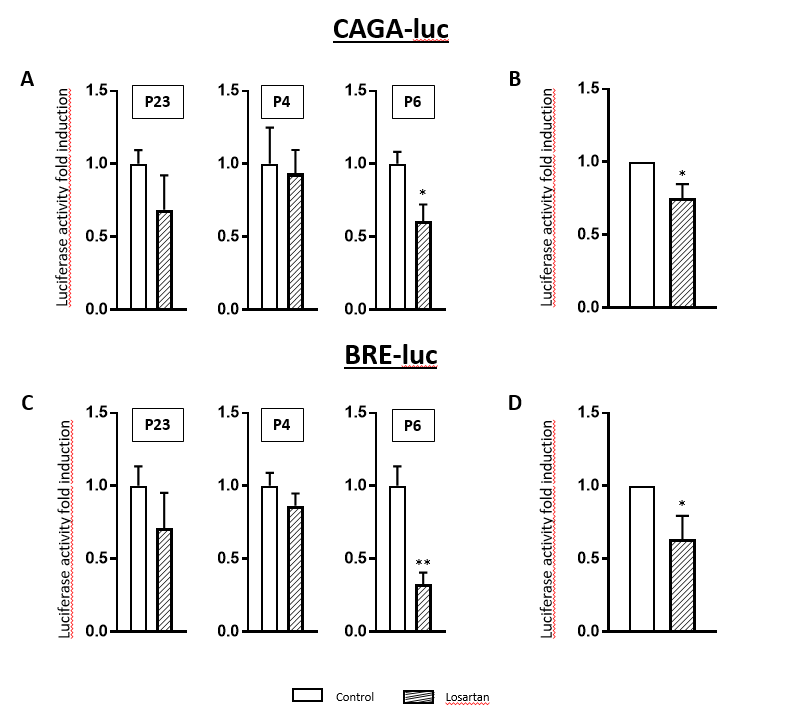


**Figure 1.** Losartan modulates TGF-β1 signalling. Human fibroblast from EB patients were transiently transfected with the promoter reporter vectors CAGA-luc (**A** and **B**) and BRE-luc (**C** and **D**). Luciferase assays were performed in three patients. Panels B and D show the mean of the fold induction in the three patients. Luciferase activity was measured in untreated cells or cells exposed to Losartan during 48 h in the presence of TGF-β1 during (**A-B**) the last 24 h or (**C–D**) 3 h. Differences were statistically significant according to the Student’s t-test. * *p* < 0.05; ** *p* < 0.01.


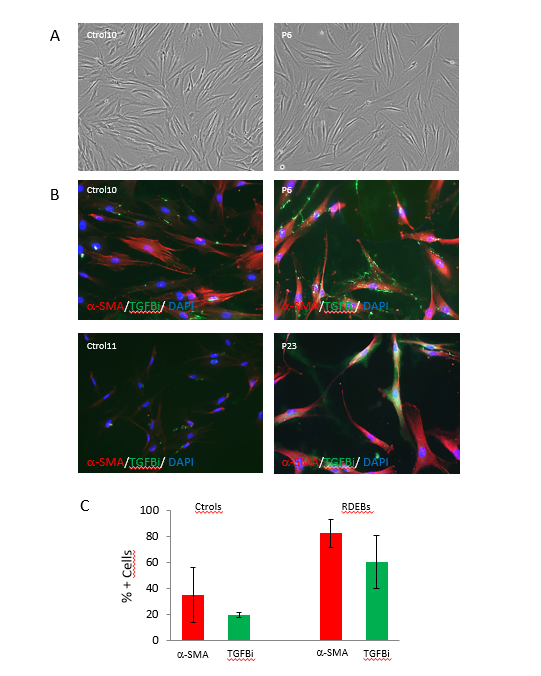


**Figure 2.** Characterization of control donor and RDEB fibroblasts in 2D culture. (**A**) Representative bright field of a control donor fibroblasts (Ctrol10) and a RDEB patient(P6) fibroblasts (Magnification: 100X). (**B**)Expression of a-SMA (red) and TGFBi ( green) in 2 different control fibroblasts (Ctrol10 and Ctrol11) and 2 different RDEB patient (P6 and p23) fibroblasts (Magnification: 200X). Cell nuclei were stained with DAPI(blue). (**C**) Percentage of positive cells for a-SMA and TGFBi in two differents fibroblasts sample of control donor (Ctrol10 and Ctrol11) and RDEB patients (P6 and p23) (10 different fields were counted for each sample).


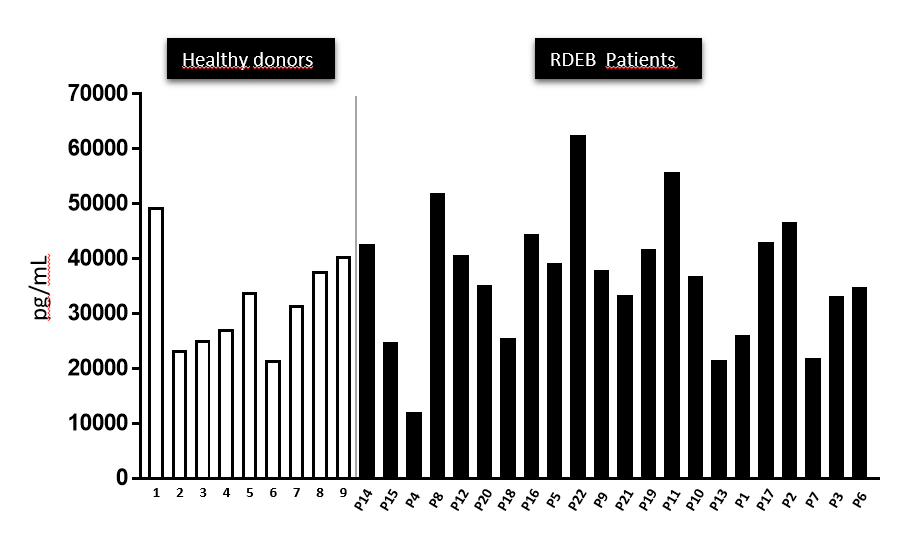


**Figure 3.** The total TGF-β1 peptide in the serum of 22 RDEB patients relative to the healthy control individuals. ELISA measurement of the total TGF-β1 peptide showing no significant differences between both groups.
